# Preemptive multimodal analgesia for gynecologic oncology patients undergoing laparotomy: a randomized controlled trial

**DOI:** 10.3389/fmed.2024.1427548

**Published:** 2024-08-29

**Authors:** Zhiyu Geng, Bojie Wang, Yan Zhang, Xin Yan, Jun Hu, Ran Cui, Linlin Song

**Affiliations:** ^1^Department of Anesthesiology, Peking University First Hospital, Beijing, China; ^2^Department of Anesthesiology, The University of Hong Kong Shen Zhen Hospital, Shen Zhen, China; ^3^Department of Obstetrics and Gynecology, Peking University First Hospital, Beijing, China

**Keywords:** gynecologic oncology, laparotomy, multimodal analgesia, postoperative pain, recovery

## Abstract

**Purpose:**

Gynecologic oncology laparotomy leads to severe postoperative pain. We aimed to evaluate the effects of preemptive multimodal analgesic regimen on postoperative opioid consumption for patients undergoing gynecologic oncology laparotomy.

**Methods:**

In this prospective, randomized clinical trial, 80 female patients scheduled for gynecologic oncology laparotomy were randomized to receive preemptive multimodal analgesia consisted of transversus abdominis plane (TAP) block, cyclooxygenase−2 inhibitors, acetaminophen and intravenous morphine patient-controlled analgesia (PCA) (Study group) or conventional analgesia with cyclooxygenase−2 inhibitors and morphine PCA (Control group). The primary outcome was morphine consumption in the first 24 h after surgery. Secondary outcomes were pain scores, nausea, vomiting, time to ambulation and flatus, length of hospital stay, satisfaction score, the 40-item Quality of Recovery score (QoR-40) and the Short-Form Health Survey (SF-36) scale.

**Results:**

Morphine consumption in the first 24 h was 6 (3–9.8) mg in the Study group and 7 (3.5–12.5) mg in the Control group (*p* = 0.222). The Study group showed lower morphine consumption up to 6 h, lower pain scores up to 48 h, and earlier time to ambulation and flatus. The global QoR-40 score at 48 h [182 (173–195) vs. 173.5 (154–185.5), *p* = 0.024], subdimension scores of physical dependence at 24 h, physical comfort and pain at 48 h were significantly improved in the Study group.

**Conclusion:**

Preemptive multimodal analgesia was not superior to conventional analgesia in reducing 24 h morphine consumption; however, it showed a significantly improved pain control and early quality of recovery thus can be recommended for gynecologic oncology patients undergoing laparotomy.

## Introduction

Gynecologic malignancy is one of the most common tumors affecting women throughout the world. Gynecologic oncology surgery can vary from minimally invasive laparoscopic surgery to major debulking procedures. Midline laparotomy often results in more severe postoperative pain than minimally invasive surgery. Since insufficient pain control may lead to chronic postsurgical pain, and the incidence and severity of chronic pain could be reduced or prevented by well-managed perioperative pain, adequate pain control is critical to patient recovery and often more challenging in the perioperative period (1, 2).

Neuraxial block with superior analgesia and better recovery of gastrointestinal function has once been considered as the gold standard of pain control after major abdominal surgery (3–5). However, perioperative venous thromboembolism prophylaxis and fast track protocols have questioned the position of epidural analgesia as a preferred analgesic technique, thus less invasive techniques including continuous wound infiltration, paravertebral and transversus abdominis plane (TAP) blocks are now widely used for abdominal surgery. Recent studies confirmed that TAP block has decreased incidence of hypotension when compared to epidural analgesia in major abdominal surgery (6, 7).

As a key component of Enhanced Recovery After Surgery (ERAS) pathway, multimodal analgesia using multiple pharmacologic agents with different analgesic mechanisms of action is recommended to improve patient’s recovery. Multimodal analgesia has been demonstrated to result in less opioid consumption and reduced length of hospital stay for different types of surgery (8–10).

TAP block resulted in reduced pain scores and opioid consumption when used as part of multimodal pain protocol in abdominal procedures (11–15). To our knowledge, there is limited evidence for the effect of preemptive TAP block-based multimodal analgesia on gynecologic oncology laparotomy (16–19). We hypothesized that preemptive TAP block-based multimodal analgesia would reduce total opioid consumption in the first 24 h compared to conventional analgesia. The primary aim of this study was to compare the total morphine consumption in the first 24 h when patient was randomized to either preemptive TAP block-based multimodal analgesia or conventional analgesia. Secondary aims included pain scores, nausea and vomiting, function recovery, length of hospital stay, satisfaction score, patient-reported quality of recovery measured using the 40-item Quality of Recovery score (QoR-40) and the Short-Form Health Survey (SF-36).

## Materials and methods

### Study design

This single-center, prospective randomized controlled trial was conducted at Peking University First Hospital, with ethical approval (No. 2019–199) provided by the Ethics Committee of Peking University First Hospital, Peking, China on 11 September 2019. The study was registered prior to patient enrollment at chictr.org.cn (ChiCTR 2,000,029,903; Principal investigator: Bojie Wang; Date of registration: February 16, 2020). Written informed consent was obtained from all patients participating in the trial. This manuscript adheres to the applicable Consolidated Standards of Reporting Trials (CONSORT) guidelines.

### Inclusion and exclusion criteria

From August 2021 to August 2022, all female patients scheduled for elective gynecologic oncology laparotomy were screened for eligibility. Eligible criteria included 18–65 years of age, and American Society of Anesthesiologists (ASA) physical status I-III. Patients with significant liver or renal disease, history of peptic ulcer or gastrointestinal bleeding, contraindications or sensitivities to any medication used in the study, recent use of analgesic drug, known history of chronic pain disorders, inability to understand how to use the patient-controlled analgesia (PCA) device or communicate with research personnel were excluded.

### Randomization and blinding

The enrolled patients were assigned to the Study group or Control group with a ratio of 1:1. Randomization was performed using a random number generated by the computer and each number sealed in an opaque envelope by a research nurse who was not involved in this study. On the day of surgery, the envelope with allocation information was delivered to the anesthetic provider. The patients and investigators involved in outcome assessing and data collection were blinded to the group allocation.

### Anesthesia technique

Following standard monitoring, general anesthesia was induced with midazolam 0.03 mg/kg, propofol 1.5–2 mg/kg, and sufentanil 0.2 μg/kg. Rocuronium 0.6 mg/kg was given to facilitate tracheal intubation. Anesthesia was maintained with continuous infusion of propofol, target-controlled infusion of remifentanil, and intermittent bolus of sufentanil and rocuronium. Mean blood pressure and heart rate were kept within 20% of the baseline values, and the bispectral index was maintained between 40 and 60. Antiemetic therapy comprised of dexamethasone 5 mg before induction and tropisetron 5 mg during wound closure. After completion of the surgery, continuous infusion of remifentanil and propofol was ceased. Residual curarization was reversed with neostigmine and atropine. After extubation, the patient was transferred to the post-anesthesia care unit (PACU) for recovery.

### Intervention

The Study group received preemptive multimodal analgesia consisted of TAP block and parecoxib before incision, acetaminophen, celecoxib, and morphine PCA after procedure. After induction, ultrasound-guided posterior TAP block was performed before incision. After negative aspiration of blood, the correct position of the needle tip was confirmed by observed distending when 1 mL of normal saline was injected. 20ml of ropivacaine 0.375% was injected on each side between the internal oblique and transversus abdominis plane under direct visualization. Intravenous (IV) parecoxib sodium 40 mg was administered before incision. Another dose of parecoxib sodium 40 mg was administered 6 h after surgery in the surgical ward. Oral acetaminophen 650 mg every 8 h and celecoxib 200 mg every 12 h were administered on postoperative day (POD) 1 and 2. The Control group received conventional analgesic protocol including parecoxib sodium 40 mg before the end of procedure and morphine PCA.

In the PACU, all patients were given a morphine IV PCA pump which was programmed to deliver 1 mg bolus on demand with 6-min lockout interval without continuous dose as part of multimodal analgesia. Pain intensity was measured with an 11-point numerical rating scale (NRS; 0 = no pain and 10 = worst imaginable pain). Additional morphine 1–2 mg bolus was administered as rescue analgesia when breakthrough pain (NRS ≥ 4) appeared despite IV PCA administration. Nausea intensity was assessed with a verbal rating scale (0 = none, 1 = mild, 2 = moderate, 3 = severe). IV metoclopramide 5 mg was administered as rescue antiemetics for severe degree of nausea or any vomiting that patients were unable to tolerate. In the surgical ward, deep vein thrombosis prophylaxis was administered with lower extremity sequential compression devices and subcutaneous low molecular heparin. At 48 h, the doses of morphine PCA and rescued morphine in PACU were recorded as the total morphine consumption.

Patient demographics included age, weight, height, type of the surgical incision, duration of surgery, and anesthetic doses were recorded. The observation period started from the end of surgery. A blinded researcher assessed the patients at PACU, 2, 6, 24 and 48 h after surgery. After discharge, patients were followed up on POD 30 to complete the SF-36 Health Survey Questionnaire. An investigator blinded to the group allocation collected all perioperative and outcome data.

### Outcomes

The primary outcome was the total morphine consumption within the first 24 h after surgery. The secondary outcomes included: (1): NRS scores at rest and during coughing; (2) Morphine consumption at other time points; (3) Nausea, vomiting, and rescued antiemetics within the first 48 h; (4) Time to first ambulation and flatus; (5) Quality of recovery assessed with QoR-40 score at 24 and 48 h; (6) Patient satisfaction (0 = totally unsatisfied, 10 = total satisfied) assessed at 48 h; (7) Quality of life measured with the SF-36 questionnaire on POD 30; (8) length of hospital stay; (9) Major postoperative complications including surgical site infection, urinary tract infection, sepsis, pneumonia, myocardial infarction, renal insufficiency, red blood transfusion, thromboembolic events, intensive care unit (ICU) admission, and requiring repeat surgery.

### Sample size calculation

Sample size calculation was based on previous studies involving TAP block-based analgesia after gynecologic laparotomy (13, 17, 18). To detect a 30% reduction in postoperative morphine consumption, the sample size was 36 patients in each group with a power of 0.80 and a 2-tailed alpha of 0.05. Considering a 10% possible dropout, we planned to recruit a total of 80 patients in the study.

### Statistical analysis

The data were analyzed with SPSS version 22.0 (IBM, United States). Continuous variables were reported as means ± standard deviation (SD) or medians [interquartile ranges (IQRs)] according to the normality of data distribution checked by the Shapiro–Wilk test. The significance of differences between groups was compared using 2 independent sample Student’s *t* test or Mann–Whitney *U* test. The differences of the medians and 95% confidence interval (CI) were estimated using the Hodges–Lehman method. Bonferroni correction was used and the *p* value was adjusted for repeated measurements. Categorical variables were described as number (percentage), and compared using Pearson’s *χ*^2^ test or Fisher’s exact test as appropriate. All *p* values were 2-sided, and *p* < 0.05 was deemed statistically significant.

## Results

**Figure 1:**
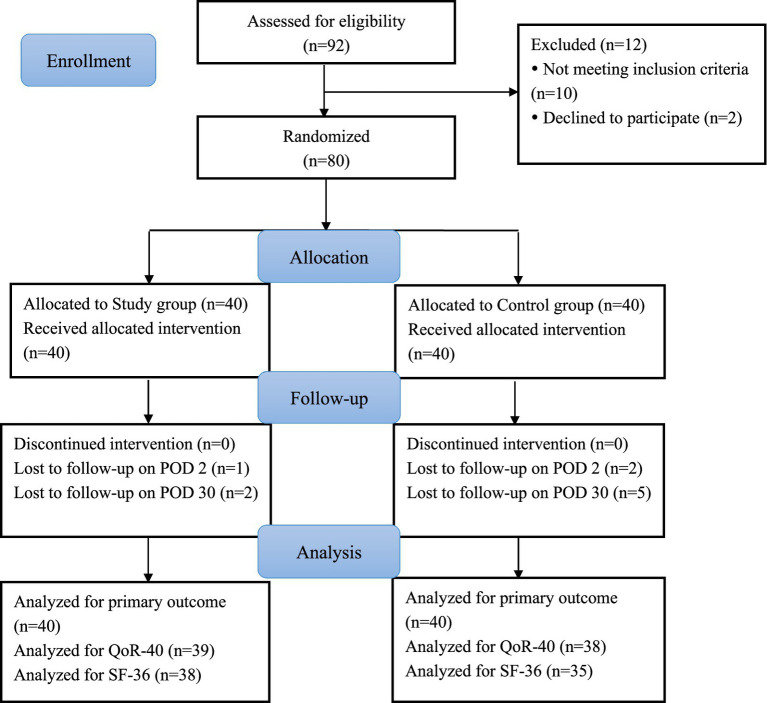
Participant flowchart.

From August 2021 to August 2022, 92 patients were assessed for eligibility. 12 patients were excluded for not meeting the inclusion criteria (*n* = 10) or declining to participate (*n* = 2). Thus, 80 patients were randomized to either the Study group or the Control group. All patients received analgesia in compliance with the protocol and were included in the intention-to-treat analysis of primary outcome. The CONSORT flow diagram is presented in Figure 1.

The two groups were similar in baseline demographics and operation characteristics. There were no significant differences in intraoperative propofol, remifentanil, or sufentanil doses between two groups (Table 1).

**Table 1:** Patient characteristics and surgical data.

Variables	Study group (*n* = 40)	Control group (*n* = 40)	*p*-value
Age (year)	49.4 ± 8.8	51.1 ± 8.3	0.389
Height (cm)	160.3 ± 6.2	158.7 ± 5.5	0.225
Weight (kg)	63.3 ± 8.5	65.2 ± 12.4	0.433
BMI (kg/m^2^)	24.7 ± 3.2	25.9 ± 4.6	0.173
ASA classification I/II/III (n)	14/25/1	15/23/2	0.798
PONV score	3.4 ± 0.6	3.2 ± 0.5	0.685
Operation (*n*/%)			0.134
Cervical cancer	26 (65%)	16 (40%)	
Endometrial cancer	7 (17.5%)	10 (25%)	
Ovarian cancer	4 (10%)	6 (15%)	
Other	3 (7.5%)	8 (20%)	
Type of incision (*n*/%)			0.890
Pfannenstiel incision	2 (5%)	2 (5%)	
Vertical incision above the umbilicus	24 (60%)	26 (65%)	
Vertical incision below the umbilicus	14 (35%)	12 (30%)	
Anesthesia time (min)	232.3 ± 65.8	234.1 ± 67.2	0.905
Operation time (min)	204.6 ± 64.2	209.7 ± 63.7	0.721
Blood loss (ml)	200 (200–400)	200 (200–375)	0.837
Intraoperative remifentanil (μg/kg/h)	5.5 (4.7–5.8)	5.2 (4.4–5.7)	0.424
Intraoperative sufentanil (μg/kg)	0.5 ± 0.1	0.5 ± 0.1	0.712

Data are presented as mean ± standard deviation, median (interquartile range), or number (%) where appropriate. ASA, American Society of Anesthesiologists; BMI, body mass index; PONV, postoperative nausea and vomiting.

Morphine consumption within the first 24 h was similar between the two groups [Study group: 6 (3–9) mg vs. Control group: 7 (3.5–12.5) mg, mean difference, −1 (95% confidence interval CI, −4 to 1) mg, *p* = 0.222]. For secondary outcomes, the Study group had significantly less morphine consumption up to 6 h [4 (2–5) mg vs. 5 (3–9) mg, mean difference, −2 (95% CI, −3 to−1) mg, *p* = 0.000], lower pain scores at rest and during coughing at any time point up to 48 h than the Control group (Table 2).

**Table 2:** Postoperative pain management and adverse events.

	Study group (*n* = 40)	Control group (*n* = 40)	Median difference (95% CI)	*p*-value
Morphine consumption, 2 h (mg)	2 (1–4)	4 (2–6.5)	-2 (−3 to−1)	**0.000** ^a^
Morphine consumption, 6 h (mg)	4 (2–5)	5 (3–9)	-2 (−3 to−1)	**0.000** ^a^
Morphine consumption, 24 h (mg)	6 (3–9.8)	7 (3.5–12.5)	−1 (−4 to 1)	0.222
NRS at PACU				
Rest	1 (1–2)	2 (2–3)	−1 (−1 to−1)	**0.000** ^b^
Coughing	3 (2–3)	3 (2.3–4)	-1 (−2 to-1)	**0.000** ^b^
NRS at 2 h				
Rest	1 (1–2)	2 (2–3)	-1 (−1 to-1)	**0.000** ^b^
Coughing	2.5 (2–3)	4 (3–5)	-1 (−2 to-1)	**0.000** ^b^
NRS at 6 h				
Rest	1 (1–2)	2 (2–3)	-1 (−1 to-1)	**0.000** ^b^
Coughing	3 (2–4)	4 (3–4)	-1 (−1 to-1)	**0.000** ^b^
NRS at 24 h				
Rest	1 (1–2)	2 (2–3)	-1 (−1 to-1)	**0.000** ^b^
Coughing	3 (2–4)	4 (3–5)	-1 (−2 to-1)	**0.000** ^b^
NRS at 48 h				
Rest	1 (1–1)	2 (1–2)	-1 (−1 to 0)	**0.000** ^b^
Coughing	2 (2–3)	3 (3–4)	-1 (−1 to-1)	**0.000** ^b^
Nausea 0-48 h (n/%)	8 (20%)	12 (30%)		0.302
Vomiting 0-48 h (n/%)	7 (17.5%)	10 (25%)		0.412
Rescue antiemetics 0-48 h (n/%)	1 (2.5%)	3 (7.5%)	1.053	0.305

Data are presented as median (interquartile range) and number (%) where appropriate. CI, confidence interval; NRS, numerical rating scale; PACU, post-anesthesia care unit. ^a^Bonferroni correction was used for 3 comparisons, *p* < 0.03 was considered statistical significance. ^b^Bonferroni correction was used for 5 comparisons, *p* < 0.01 was considered statistical significance. Bold value is used to highlight significant values.

The global QoR-40 score at 48 h was significantly higher in the Study group compared to the Control group [182 (173–195) vs. 174 (154–186), *p* = 0.024]. Among five dimensions of QoR-40, physical dependence at 24 h [24 (21–25) vs. 22 (18–25), *p* = 0.019], physical comfort [55 (52–59) vs. 51 (44–55), *p* = 0.003] and pain at 48 h [32 (30–33) vs. 31 (28–32), *p* = 0.023] were significantly improved in the Study group (Table 3).

**Table 3:** Functional recovery parameters.

	Study group (*n* = 39)	Control group (*n* = 38)	Median difference (95% CI)	*p*-value
Global QoR-40 score				
24 h	182 (167–191)	175 (160.3–83.8)	6 (−2 to 13)	0.122
48 h	182 (173–195)	173.5 (154–185.5)	10 (1 to 18)	**0.024**
Emotional state				
24 h	38 (33–43)	38 (34–42)	0 (−2 to 3)	0.672
48 h	41 (35–44)	36.5 (33.3–41)	2 (0 to 5)	0.062
Physical comfort				
24 h	53 (50–57)	51 (47–55.8)	2 (−1 to 5)	0.202
48 h	55 (52–59)	51.5 (44–54.8)	5 (2 to 8)	**0.003**
Psychological support				
24 h	34 (32–35)	34 (31–35)	0 (0 to 1)	0.497
48 h	35 (31–35)	34 (29.8–35)	0 (0 to 1)	0.682
Physical independence				
24 h	24 (21–25)	22 (18.3–25)	2 (0 to 3)	**0.019**
48 h	24 (20–25)	21.5 (17–24.8)	1 (0 to 3)	0.094
Pain				
24 h	31 (30–33)	31 (28.3–32)	1 (0 to 2)	0.158
48 h	32 (30–33)	30.5 (28–32)	1 (0 to 3)	**0.023**
Time to ambulation (h)	20 (17–24)	21 (19–35.5)	−3.5 (−8 to-0.5)	**0.021**
Time to flatus (h)	34 (24–47)	48 (39.5–62)	−13 (−20 to−5)	**0.001**
Complications in-hospital (n/%)	5 (12.5%)	9 (22.5%)	1.385	0.239
Patient satisfaction score	10 (9–10)	10 (9–10)	0 (0 to 0)	0.123
Length of hospital day (d)	8.5 (7–14)	9 (7–10)	0 (−1 to 2)	0.985

Data are presented as median (interquartile range) and number (%) where appropriate. QoR-40, Quality of Recovery 40; CI, confidence interval. Bold value is used to highlight significant values.

Patients in the Study group had shorter time to ambulation [20 (17–24) h vs. 21 (19–35.5) h, *p* = 0.021] and flatus [34 (24–47) h vs. 48 (39.5–62) h, *p* = 0.001] than the Control group.

No difference in global QoR-40 score at 24 h and SF-36 scores on POD 30 was observed (Tables 3, 4). Nausea, vomiting, need for rescue antiemetic, complications in-hospital, and length of hospital stay were similar between groups. Patient satisfaction was equally high in both groups.

**Table 4:** SF-36 scores on POD 30.

	Study group (*n* = 38)	Control group (*n* = 35)	Mean or median difference (95% CI)	*p*-value
Global SF-36 score	443 ± 120	445 ± 116	27.7 (−57.7 to 52.8)	0.930
Physical functioning	60 (50–71)	65 (55–80)	−5 (−15 to 5)	0.181
Role-physical	0 (0–25)	0 (0–25)	0 (0 to 0)	0.916
Bodily pain	62 (52–73)	62 (51–62)	0 (0 to 11)	0.376
General health	67 (57–87)	65 (57–72)	5 (−5 to 13)	0.349
Vitality	67 ± 18	66 ± 17	0.8 (−7.3 to 8.9)	0.197
Social functioning	63 (47–75)	63 (63–75)	0 (−12.5 to 12.5)	0.569
Role-emotional	33 (0–67)	33 (0–67)	0 (0 to 0)	0.798
Mental health	74 ± 17	73 ± 16	1.5 (−6.3 to 9.3)	0.702
Health transition	50 (25–75)	50 (25–75)	0 (−25 to 0)	0.527

Data are presented as mean ± standard deviation and median (interquartile range) where appropriate. SF-36, 36-item of Short Form Health Survey; POD postoperative day; CI confidence interval.

## Discussion

In this randomized controlled trial, patients who received preemptive TAP block-based multimodal analgesia did not have lower 24-h morphine consumption than the patients who received conventional analgesia. However, morphine consumption in the first 6 h, pain scores up to 48 h, and in-hospital functional recovery were significantly improved in the preemptive TAP block-based multimodal analgesic group.

Pain after abdominal surgery is caused by the incision (somatic pain) and trauma to intra-abdominal structures (visceral pain). TAP block is performed to anaesthetize the branches of T6 to L1 sensory nerve roots that innervate the anterior abdominal wall (20). Abdominal wall pain from laparotomy incision can be effectively prevented by the TAP block, and visceral pain can be treated by systemic analgesia consisted of acetaminophen, nonsteroidal anti-inflammatory drugs (NSAIDs), and opioids.

For patients undergoing hysterectomy for benign disease, TAP block has shown significant reduction in pain scores and opioid consumption, and its effect is even more profound if administered before rather than after surgical incision (21). Gasanova et al. (13) showed that for patients undergoing total abdominal hysterectomy, pain on coughing was less variability in TAP block with acetaminophen and NSAID group compared to only TAP block or only acetaminophen and NSAID group. Røjskjaer et al. (22) demonstrated lower pain scores in the early postoperatively period when adding TAP block to a multimodal analgesic regimen consisted of acetaminophen, ibuprofen, dexamethasone, and celecoxib.

Gynecologic oncology laparotomy often involves a large midline vertical incision and considerable visceral disruption thus could induce more complex surgical stress response and severe postoperative pain. However, previous studies investigating TAP block on opioid requirement or pain intensity failed to show any benefit effects in this setting.

In a retrospective study of 120 patients who underwent extensive surgical resection for ovarian cancer, there was no significant difference in opioid consumption within the first 24 h between TAP block group (15.83 mg of morphine equivalent daily dose, IQR 10–34) and no TAP block group (18.75 mg of morphine equivalent daily dose, IQR 7.5–31) (23). Griffiths et al. (18) demonstrated that TAP block performed at the end of gynecologic cancer surgery conferred no benefit in addition to multimodal analgesia. Postoperative morphine consumption at 2 h or 24 h were similar between the placebo and TAP groups. Hotujec et al. (19) showed that preoperative TAP block did not reduce opioid use or postoperative pain scores for gynecologic malignancy patients underwent robotic-assisted laparoscopic surgery. The TAP block group used a mean of 64.9 mg morphine in the first 24 h compared to 69.3 mg for controls.

Preemptive analgesia is an analgesia intervention given before noxious stimulus arises to prevent peripheral and central sensitization caused by incisional and inflammatory injuries. Preemptive analgesia is thought to reduce postoperative pain and hyperalgesia by decreasing production of proinflammatory cytokines (24). A recent meta-analysis indicated that preemptive analgesia reduced postoperative pain, opioid consumption, postoperative nausea or vomiting, and delayed rescue analgesia (25). NSAIDs and cyclooxygenase-2 (COX-2) inhibitors were recommended as preemptive pain medication prior to abdominal hysterectomy to decrease narcotic requirements and improve patient pain assessment and satisfaction scores (26).

In our present study, TAP block combined with intravenous parecoxib was given prior to incision as preemptive multimodal analgesic regimen. Although morphine consumption was only decreased up to 6 h, pain scores at rest and with coughing were significantly improved throughout the 48 h postoperatively in the Study group.

As an essential component of ERAS pathway, multimodal analgesic regimen aims to facilitate patient’s recovery and return to baseline function other than provide adequate pain relief. The QoR-40 score is a patient-reported global measure of quality of recovery and is recommended to be used as standardized endpoints in perioperative clinical trials (27, 28). The SF-36 health survey is a multidimensional measure used to assess health-related quality of life after surgery. The high score indicates a more favorable health state (29). A poor-quality recovery measured by lower QoR-40 score in the early postoperative period can predict a poor quality of life measured by the SF-36 at 3 months after surgery (30).

Our result showed that patients in the Study group had higher global QoR-40 score at 48 h, subdimension score of physical dependence at 24 h, physical comfort and pain at 48 h, confirming a higher recovery quality in the postoperative period. Adequate pain controls up to postoperative 48 h may contribute to improved functional recovery and patients in the Study group had earlier mobilization and shorter time to flatus in the early postoperative period.

Our study has several limitations. First, the sensory blockade level of TAP block was not assessed before incision since it was performed after induction. Nevertheless, we performed the block under real-time ultrasound guidance to obtain proper spread of the solution in the target plane and adequate analgesia was provided in the Study group. Second, the study size was relatively small and there may be other factors that cannot be controlled adequately, such as patient frailty and surgical complexity score, which are important factors associated with postoperative complications (31, 32). Finally, we did not design this study to analyze the chronic postsurgical pain. Further study is needed to investigate whether multimodal analgesia could reduce or prevent chronic postsurgical pain after gynecologic oncology laparotomy.

## Conclusion

Our results demonstrated preemptive multimodal analgesia was not superior to conventional multimodal analgesia in reducing 24-h morphine consumption; however, it showed a significantly improved pain control and early quality of recovery thus can be recommended for gynecologic oncology patients undergoing laparotomy.

## Data availability statement

The raw data supporting the conclusions of this article will be made available by the authors, without undue reservation.

## Ethics statement

The studies involving humans were approved by the Ethics Committee of Peking University First Hospital. The studies were conducted in accordance with the local legislation and institutional requirements. The participants provided their written informed consent to participate in this study.

## Author contributions

ZG: Conceptualization, Formal analysis, Writing – original draft. BW: Conceptualization, Data curation, Funding acquisition, Writing – review & editing. YZ: Investigation, Project administration, Resources, Writing – review & editing. XY: Project administration, Resources, Writing – review & editing. JH: Project administration, Resources, Writing – review & editing. RC: Supervision, Visualization, Writing – review & editing. LS: Software, Validation, Writing – review & editing.
